# Prenatal Exposure to Perfluorooctanoate (PFOA) and Perfluorooctanesulfonate (PFOS) and Maternally Reported Developmental Milestones in Infancy

**DOI:** 10.1289/ehp.11277

**Published:** 2008-06-04

**Authors:** Chunyuan Fei, Joseph K. McLaughlin, Loren Lipworth, Jørn Olsen

**Affiliations:** 1 Department of Epidemiology, University of California, Los Angeles, California, USA; 2 International Epidemiology Institute, Rockville, Maryland, USA; 3 Vanderbilt University Medical Center, Vanderbilt-Ingram Cancer Center, Nashville, Tennessee, USA; 4 Institute of Public Health, University of Aarhus, Aarhus, Denmark

**Keywords:** maternal blood, mental developmental milestones, motor developmental milestones, PFOA, PFOS

## Abstract

**Background:**

Perfluorooctanesulfonate (PFOS) and perfluorooctanoate (PFOA) are fluorinated organic compounds present in the general population at low concentrations. Animal studies have shown that they may affect neuromuscular development at high concentrations.

**Objectives:**

We investigated the association between plasma levels of PFOS and PFOA in pregnant women and motor and mental developmental milestones of their children.

**Methods:**

We randomly selected 1,400 pairs of pregnant women and their children from the Danish National Birth Cohort. PFOS and PFOA were measured in maternal blood samples taken in early pregnancy. Apgar score was abstracted from the National Hospital Discharge Register in Denmark. Developmental milestones were reported by mothers using highly structured questionnaires when the children were around 6 months and 18 months of age.

**Results:**

Mothers who had higher levels of PFOA and PFOS gave birth to children who had similar Apgar scores and reached virtually all of the development milestones at the same time as children born to mothers with lower exposure levels. Children who were born to mothers with higher PFOS levels were slightly more likely to start sitting without support at a later age.

**Conclusion:**

We found no convincing associations between developmental milestones in early childhood and levels of PFOA or PFOS as measured in maternal plasma early in pregnancy.

Perfluorooctanesulfonate (PFOS) and perfluorooctanoate (PFOA), fully fluorinated organic compounds, have been widely used in consumer products (carpets, furniture, household cleaner, fabrics, and paper products), and manufacturing processes (industrial surfactants and emulsifiers). The widespread distribution and persistence of these compounds in humans and the environment warrant study of their potential health impact early in life when susceptibility may be highest (Apelberg et al. 2007; Fei et al. 2007).

PFOA and PFOS cross the placental barrier during pregnancy (Apelberg et al. 2007; Fei et al. 2007; Inoue et al. 2004; Jin et al. 2004; Midasch et al. 2007; Tittlemier et al. 2004). They are present in cord blood at concentrations 1.5–3.5 times lower than those found in maternal blood (Fei et al. 2007; Inoue et al. 2004), and they may reduce fetal growth in animals and humans (Apelberg et al. 2007; Butenhoff et al. 2004; Fei et al. 2007, 2008; Grice et al. 2007; Lau et al. 2006; Luebker et al. 2005a, 2005b; Thibodeaux et al. 2003). There are no data on their developmental neurotoxic effects in humans, but exposure of animals to PFOS or PFOA during pregnancy may lead to a decrease in motor function (Fuentes et al. 2007a, 2007b; Johansson et al. 2008) and delayed learning (Fuentes et al. 2007a), as indicated by some animal studies but not all (Lau et al. 2003). PFOS or PFOA were also associated with decreased viability, accelerated or delayed sexual maturation, and delays in eye opening and other developmental end points in rats (Austin et al. 2003; Butenhoff et al. 2004; Lau et al. 2003; Luebker et al. 2005a, 2005b) or mice (Lau et al. 2003, 2006).

In this study we examined exposure to PFOA or PFOS and neurodevelopmental or musculoskeletal development using data on achievement of developmental milestones in early childhood from the Danish National Birth Cohort (DNBC) (Olsen et al. 2001).

## Materials and Methods

The DNBC recruited women in early pregnancy from March 1996 to November 2002 through their general practitioners (GPs) (Olsen et al. 2001). All pregnant women who spoke Danish well enough and intended to carry the pregnancy to term were considered eligible for the study. Approximately 50% of GPs took part in the study, and about 60% of the pregnant women accepted the invitation from their GPs (Olsen et al. 2001). Study data were collected by computer-assisted telephone interviews at approximately 12 and 30 weeks of gestation, and when children reached the ages of approximately 6 months and 18 months. Blood samples were drawn from the mother twice during pregnancy and from the child (umbilical cord) shortly after birth. Only blood samples taken in the first trimester were used in this analysis.

Among 43,045 women who met our sampling criteria, we randomly selected 1,400 blood samples from the mothers within the DNBC. PFOA and PFOS concentrations were analyzed by high-performance liquid chromatography/tandem mass spectrometry in the 3M Toxicology Laboratory (St. Paul, MN, USA). Detailed sampling and laboratory methods have been previously published (Fei et al. 2007). Written informed consent was obtained from all participants at recruitment, and the Office for Protection of Research Subjects at University of California, Los Angeles, and the Danish Data Protection Agency approved the study protocol.

We obtained data on Apgar scores by linking cohort members to the National Hospital Discharge Register (Copenhagen, Denmark) through use of the unique personal identification number assigned to all Danish residents at birth or immigration. The Apgar score was recorded as the sum of 0–2 points (in total 0–10 points) from five characteristics (heart rate, respiratory effort, reflex irritability, muscle tone, and skin color) (Apgar 1953). Midwives assessed Apgar score 5 min after birth following standardized procedures. Motor and mental development of infants at 6 months and 18 months of age was assessed based on mothers’ self-reports. Mothers were asked questions developed by an experienced child neuropsychologist about developmental milestones within the mental and motor domains. The motor domains assessed gross and fine motor functioning. The mental domains assessed the child’s attention and cognitive functions, language development, and social-personal development. For instance, at the 18-month assessment, nine questions had a yes/no answer (e.g., “can he/she take off socks and shoes when you ask him/her to do so?”). Another three questions required more detailed responses, including *a*) “how old was he/she when he/she could sit with no support?”; *b*) “how old was he/she when he/she could walk with no support?”; and *c*) “approximately how many things can he/she mention by name?”, for which the mother could choose among five categories (0–10, 11–25, 26–100, 101–300, and ≥300 words), with one additional category of free text (Olsen et al. 2001). (The questionnaires are available at http://www.bsmb.dk)

Subjects were divided *a priori* into four categories on the basis of quartiles of maternal PFOA and PFOS exposure during pregnancy. Logistic regression was used for dichotomized outcomes. Ordinal logistic regression was used for outcomes on an ordinal scale (Apgar score, number of things mentioned by name). Apgar score was also dichotomized at the cut point of 10 because of few children with low values (i.e., only nine infants had Apgar score < 7, above which is considered in the normal range, and two infants had Apgar score < 3, which is considered critically low).

We used Cox survival models to compare the age at which the child could sit or walk without support. The time from birth to the age when children (in months or in weeks) started sitting or walking alone was used as the underlying time variable. We also repeated the analyses by combining the data on “sitting” from both postnatal interviews. If their mothers answered “do not know” to the question of when the child started sitting (*n* = 82) at the 18-month interview and also reported their children could not sit at the 6-month interview, we consider these censored (*n* = 66) at the 6-month interview. Similar analyses were repeated for the outcome of when the child started walking, and those mothers who answered “do not know” at that time were treated as censoring events at the 18-month interview (*n* = 15). Hazard ratios (HRs) < 1 indicate delayed motor development (e.g., the child started sitting or walking at a later age).

Maternal age, maternal occupational and educational status, parity, prepregnancy body mass index (BMI), smoking and alcohol consumption during pregnancy, gestational weeks at blood drawing, child’s sex, child’s age at mother’s interview, breast-feeding after the child turned 6 months of age (from data at the 18-month interview), out-of-home day care (i.e., in day nursery or regular child care), how many hours the mother spent with the child every day (assessed at the 18-month interview), and home density (the total number of rooms divided by the total number of persons in the house) were considered as potential confounders (the categories are presented in Table 1). Based on prior knowledge of potential determinants of early childhood development and/or change-in-estimate principles, all mental and motor outcomes, for consistency, were adjusted for covariates that satisfied these criteria. In the analyses of Apgar score, we did not include the following variables in the model: child’s age at interview, breast-feeding, out-of-home child care, how many hours the mother spent with the child every day, and home density. Inclusion of gestational age at birth in the multivariate models did not materially change the estimates of either PFOA or PFOS. Paternal education and occupation were also evaluated, but they did not change the estimates for any of the outcomes, and therefore were not included in the final models.

We also restricted our analyses to children between 5 and 7 months of age at the time of the 6-month interview (*n* = 1,336), and children between 18 and 20 months of age in the assessment of the 18-month milestones (*n* = 1,255). All statistical analyses were performed using SAS statistical software (version 9.1.3; SAS Institute Inc., Cary, NC, USA).

## Results

Table 1 shows maternal PFOA and PFOS levels according to characteristics of the mothers and infants. Most of the mothers (90%) were > 25 years of age at delivery (Table 1), and almost half of the women were having their first baby. The levels of maternal PFOS and PFOA decreased with increasing parity and with decreasing prepregnancy BMI. Mothers whose children were being breast-fed at 6 months (36%) had significantly lower levels of PFOA and PFOS during pregnancy. At the 18-month interview, there was no significant difference in average infant age between exposure quartiles, although children in the higher PFOA group were slightly younger. At the 6-month interview, children whose mothers had higher PFOA levels were significantly older than those having lower levels.

In this sample only 0.65% of the newborns had an Apgar score < 7 at birth, and about 93% had an Apgar score equal to 10. Using ordinal regression did not show any significant association between perfluorinated chemical (PFC) level and Apgar score. When the dichotomized Apgar score using a cut-point of 10 was evaluated, the proportion of newborns with an Apgar score < 10 slightly increased with increasing PFOA levels, with 5.5% in the first quartile and 8.4% in the fourth quartile. Similarly, the proportion in the fourth quartile of PFOS levels (9.8%) was higher than the lower three quartiles (range, 5.2–7.2%). After adjustment for potential confounders, none of these associations were statistically significant; the odds ratio (OR) for Apgar score < 10 was 1.20 [95% confidence interval (CI), 0.67–2.14] and 1.14 (95% CI, 0.57–2.25) when comparing the fourth quartile with the first quartile of PFOS and PFOA, respectively.

At the 18-month interview, the median age of children was 19.0 months (range, 17–25), and 90% of the children were between 18 and 20 months. The age at which children could sit without support ranged between 3 and 15 months (median = 6), and the age at which they could walk without support ranged between 8 and 19 months (median = 12). No significant associations were observed between PFOA or PFOS levels and the age when children could walk (Tables 2 and 3). However, children whose mothers had higher PFOS levels were more likely to start sitting without support at a later age; the adjusted HRs were 0.85 (95% CI, 0.72–0.99) for the third quartile and 0.86 (95% CI, 0.73–1.01) for the fourth quartile (*p* for trend = 0.041) compared with the first quartile. Results did not change when the mothers who answered “do not know” were entered as censored data.

We observed no association between maternal plasma PFOA or PFOS levels and most of the outcomes assessed at the 18-month interview. Women with higher PFOS levels, but not women with higher PFOA levels, were more likely to have babies who “did not use word-like sounds to tell what he/she wants” (OR for the fourth quartile of PFOS versus the first quartile = 2.93; 95% CI, 1.00–8.56; *p* for trend = 0.04). The opposite association with PFOS levels was observed for the item of “did not use sentences of two words” (*p* for trend = 0.05). In the analysis of “did not make marks on table or paper,” all the crude ORs for PFOA were above the null, but the direction was reversed after adjustment for the potential confounders. Mutual adjustment for PFOA and PFOS did not influence the ORs in any consistent way (data not shown). The analyses of data from the 6-month interview did not show any association between PFC exposure during pregnancy and motor or mental development.

## Discussion

We observed no convincing association between levels of PFOA or PFOS as measured in maternal plasma early in pregnancy and motor or mental development in early childhood. PFOS was weakly associated with a later age of being able to sit alone and to “use word-like sounds to tell what he/she wants,” but no associations were observed for other end points in the same domains or with PFOA exposure.

To our knowledge, this is the first study to investigate prenatal PFC exposure and developmental milestones in humans during the first 18 months of life. We used prospectively collected data on PFC levels in maternal plasma drawn early in pregnancy, and the exposure information was not known at the time of the interviews. The risk of differential recall bias is therefore very minimal, but some nondifferential misclassification is unavoidable, although structured questionnaires on children’s developmental milestones were administered by specially trained interviewers. All the milestones are age-dependent, and the age of the child at interview is therefore a potential source of bias that we attempted to avoid by including this variable in the statistical models. To further examine the extent of this source of bias, we restricted the analyses to those 5–6 months of age at the 6-month interview, as well as those 18–20 months of age at the 18-month interview, and found similar results to those presented (data not shown).

We used PFC levels measured in maternal blood early in pregnancy as the measure of fetal exposure, but it is not clear to what extent these measures reflect critical fetal brain exposures throughout pregnancy and in early childhood. Our data showed PFOS and PFOA levels in the first trimester were highly correlated with measurements taken in the second trimester and in cord blood values (Fei et al. 2007). Children can also be exposed through breast milk or by contact with products containing PFOA and PFOS. Analyses of breast milk in Swedish women (Karrman et al. 2007) showed that breast-milk concentration of PFOS was strongly correlated with increasing maternal serum concentration, but PFOA was less frequently detected in breast milk. Children have been shown to have higher concentrations of PFOS and PFOA than adults (Olsen et al. 2003). Our data also showed that home environment variables, including house density, number of hours the mother stayed with the child, and out-of-home child care were also significantly correlated with maternal PFC levels. It is difficult to predict how PFC exposure in childhood through breast-feeding or other sources may have influenced the results, but residual confounding by, or interactions with, childhood exposure variables cannot be ruled out.

PFOA and PFOS levels were correlated to some extent (*r* = 0.62), but mutual adjustment did not increase or decrease the ORs in any consistent way. We presented ORs that were not mutually adjusted, because this adjustment is likely to be inappropriate due to multi-collinearity between the two chemicals. Similarly, PFOA and PFOS levels were strongly related to parity in this data set (Fei et al. 2007), with higher levels observed in women having their first child. Parity therefore had a strong confounding influence in our data set, perhaps reflecting the impact of older siblings on various developmental milestones of younger siblings.

Animal studies have indicated that PFOA or PFOS may interfere with normal neuromuscular development by inhibiting choline acetyltransferase (ChAT) activity (Johansson et al. 2008; Lau et al. 2003) or by disturbing lipid metabolism (U.S. EPA 2006). ChAT activity is involved in many behavioral phenomena and cognitive functions, but it has been found slightly reduced only in the pre-frontal cortex of rat pups exposed *in utero* to PFOS, not in the hippocampus (Lau et al. 2003). PFOA is a potent peroxisome proliferator–activated receptor (PPAR) agonist that can regulate lipid metabolism in humans. PPAR-mediated effects on nervous system structure and function could be another possible mechanism (U.S. EPA 2006), given the prevalence of PPAR receptors in the brain.

In conclusion, we found little evidence to support influence of prenatal PFOA and PFOS levels on motor or mental developmental milestones in early childhood at plasma concentrations that have been reported in the general population. Because some animal data show an association, albeit at administered doses that would result in body burdens orders of magnitude higher than found in the general population, a larger study with more sensitive measures of early childhood development would be needed to rule out, with confidence, any subtler adverse effect.

## Figures and Tables

**Table 1:** Plasma concentrations of PFOS and PFOA by characteristics of study subjects.

Characteristic^a^	No. (%) (*n* = 1,400)	PFOS (ng/mL) (mean ± SD)	PFOA (ng/mL) (mean ± SD)
Maternal age at delivery (years)			
< 25	118 (8.4)	38.6 ± 12.0	6.2 ± 2.1
25–29	547 (39.1)	36.8 ± 12.8	6.0 ± 2.8
30–34	504 (36.0)	33.9 ± 13.2	5.2 ± 2.2
≥ 35	230 (16.4)	33.0 ± 12.7	5.1 ± 2.4
Maternal education			
Lower	108 (8.0)	36.5 ± 13.3	5.6 ± 2.8
Middle	375 (27.8)	38.4 ± 13.0	5.7 ± 2.4
Higher	866 (64.2)	33.9 ± 12.7	5.5 ± 2.3
Maternal occupation			
Manager/professional	227 (16.2)	31.7 ± 12.0	5.3 ± 2.4
Technician	355 (25.4)	35.6 ± 12.8	5.7 ± 2.3
Service/sales worker	478 (34.2)	36.8 ± 12.8	5.7 ± 2.8
Industrial worker	93 (6.6)	39.2 ± 13.7	6.4 ± 2.6
Student	118 (8.4)	34.1 ± 14.6	5.7 ± 2.4
Unemployed	128 (9.2)	33.3 ± 12.1	4.7 ± 2.2
Parity			
0	625 (44.7)	37.67 ± 13.04	6.65 ± 2.67
1	508 (36.3)	33.25 ± 12.73	4.75 ± 1.94
≥ 2	266 (19.0)	33.47 ± 12.47	4.65 ± 2.24
Prepregnancy BMI (kg/m^2^)			
< 18.5	58 (4.2)	33.1 ± 14.3	5.2 ± 2.2
18.5–24.9	905 (66.2)	34.6 ± 12.9	5.5 ± 2.6
25.0–29.9	299 (21.9)	36.3 ± 12.0	5.6 ± 2.3
≥ 30.0	105 (7.7)	39.3 ± 14.4	6.1 ± 2.7
Smoking during pregnancy			
Nonsmoker	1,052 (75.1)	35.7 ± 13.3	5.6 ± 2.6
Quit smoking	131 (9.4)	33.9 ± 11.6	5.8 ± 2.2
1–9 cigarettes/day	109 (7.8)	35.5 ± 12.7	5.8 ± 2.6
≥ 10 cigarettes/day	108 (7.7)	32.5 ± 11.9	4.9 ± 1.9
Alcohol consumption during pregnancy			
Nondrinker	771 (55.79)	35.4 ± 13.1	5.6 ± 2.3
0.5 drinks/week	217 (15.70)	36.2 ± 12.5	5.8 ± 2.5
1–1.5 drinks/week	226 (16.35)	35.2 ± 13.1	5.6 ± 3.3
≥ 2 drinks/week	168 (12.16)	33.4 ± 12.7	5.3 ± 2.4
House density			
< 1 room/person	350 (25.0)	31.9 ± 12.2	5.2 ± 2.3
1 room/person	426 (30.5)	36.4 ± 13.4	5.6 ± 2.3
1–1.5 rooms/person	354 (25.3)	36.0 ± 13.5	5.6 ± 2.5
≥ 1.5 rooms/person	269 (19.2)	36.9 ± 12.0	6.0 ± 3.1
Sex			
Female	690 (49.3)	35.3 ± 13.0	5.5 ± 2.4
Male	710 (50.7)	35.2 ± 12.9	5.6 ± 2.7
Breast-feeding at child’s age 6 months			
Yes	501 (35.8)	34.0 ± 13.1	5.4 ± 2.6
No	898 (64.2)	37.5 ± 12.8	6.0 ± 2.3
How many hours together with mother per day			
≤1 hr/day	287 (20.5)	35.5 ± 13.5	5.6 ± 2.2
1–4 hr/day	190 (13.6)	37.0 ± 12.2	6.0 ± 2.4
4–5 hr/day	281 (20.1)	35.4 ± 13.7	5.6 ± 2.7
5–7 hr/day	349 (24.9)	35.0 ± 12.8	5.6 ± 2.9
≥ 8 hr/day	292 (20.9)	34.0 ± 12.5	5.2 ± 2.4
Out-of-home child care			
Yes	1,229 (87.8)	35.5 ± 13.0	5.7 ± 2.3
No	170 (12.2)	33.7 ± 12.6	5.0 ± 2.6

a Missing data: maternal age (1), maternal education (50), prepregnancy BMI (33), alcohol consumption during pregnancy (18).

**Table 2:** HRs and ORs (95% CIs) for developmental milestones at the 18-month interview according to maternal PFOA level (ng/mL) in quartiles.

	< LLOQ–3.91	3.91–5.20		5.21–6.96		6.97–41.50		
Developmental milestone	No. (%)	No. (%)	HR or OR	No. (%)	HR or OR	No. (%)	HR or OR	*p* for trend
Gross motor								
Sit without support^a^,^b^ (*n* = 1,318)	Reference		0.94 (0.80–1.11)		0.91 (0.77–1.07)		0.89 (0.75–1.06)	0.191
Walk without support^a^,^b^ (*n* = 1,385)	Reference		1.10 (0.94–1.28)		1.04 (0.88–1.22)		0.94 (0.80–1.12)	0.326
Did not go up stairs with support^c^ (*n* = 1,365)	12 (3.57)	12 (3.49)	1.14 (0.47–2.80)	11 (3.22)	1.09 (0.42–2.80)	11 (3.20)	1.16 (0.42–3.21)	0.814
Fine motor^c^								
Did not take off socks and shoes when asked to do so (*n* = 1,381)	54 (15.61)	62 (17.97)	1.08 (0.70–1.65)	69 (20.00)	1.18 (0.77–1.83)	48 (13.87)	0.68 (0.42–1.11)	0.175
Did not drink from an ordinary cup without help^d^ (*n* = 1,399)	8 (2.29)	4 (1.15)	0.49 (0.15–1.66)	3 (0.85)	0.37 (0.10–1.40)	4 (1.14)	0.49 (0.15–1.65)	0.183
Attention^c^								
Was not occupied alone with the same thing for at least 15 min (*n* = 1,381)	60 (17.34)	63 (18.37)	0.85 (0.56–1.29)	62 (17.87)	0.75 (0.49–1.16)	65 (18.79)	0.76 (0.48–1.18)	0.199
Cognition^c^								
Did not bring things when told to^d^ (*n* = 1,380)	6 (1.75)	1 (0.29)	0.16 (0.02–1.37)	6 (1.72)	0.98 (0.32–3.09)	3 (0.86)	0.49 (0.12–1.97)	0.637
Did not make marks on table or paper (*n* = 1,375)	13 (3.74)	21 (6.02)	1.08 (0.50–2.34)	16 (4.58)	0.59 (0.26–1.36)	21 (6.03)	0.57 (0.25–1.32)	0.070
Did not turn the picture right when look in a book (*n* = 1,329)	181 (55.02)	169 (51.68)	0.88 (0.64–1.23)	159 (47.32)	0.73 (0.52–1.02)	168 (49.70)	0.82 (0.58–1.17)	0.181
Language^c^								
Did not use word-like sounds to tell what he/she wants (*n* = 1,380)	11 (3.15)	8 (2.29)	0.65 (0.24–1.77)	7 (1.99)	0.68 (0.23–1.96)	11 (3.14)	1.37 (0.50–3.77)	0.539
No. of things he/she can mention by name^e^ (*n* = 1,397)	Reference		0.88 (0.64–1.21)		0.86 (0.62–1.20)		0.81 (0.57–1.14)	0.254
Did not use sentences of two words (*n* = 1,357)	98 (68.53)	93 (61.18)	0.93 (0.67–1.29)	100 (62.11)	0.94 (0.67–1.32)	100 (59.52)	0.80 (0.56–1.14)	0.253

LLOQ, lower limit of quantitation (1 ng/mL).

a HRs: time to event: the time from birth to the age when children (in months) can start sitting or walking alone.

b Adjusted for maternal age, parity, smoking, and alcohol drinking during pregnancy, maternal education, maternal occupation, prepregnancy BMI, breast-feeding at child’s age 6 months, gestational weeks at blood drawing, out-of-home child care, how many hours together with mother per day, home density (rooms/person), sex.

c All the same covariates adjusted for the outcome of “sit” and “walk” plus child’s age at interview.

d Crude ORs because of sparse data.

e Ordinal logistic regression.

**Table 3:** HRs and ORs (95% CIs) for developmental milestones at the 18-month interview according to maternal PFOS level (ng/mL) in quartiles.

	6.4–26.0	26.1–33.3		33.4–43.2		43.3–106.7		
Developmental milestone	No. (%)	No. (%)	HR or OR	No. (%)	HR or OR	No. (%)	HR or OR	*p* for trend
Gross motor								
Sit without support^a^,^b^ (*n* = 1,318)	Reference		0.93 (0.79–1.08)		0.85 (0.72–0.99)		0.86 (0.73–1.01)	0.041
Walk without support^a^,^b^ (*n* = 1,385)	Reference		1.07 (0.92–1.24)		0.99 (0.85–1.15)		0.91 (0.78–1.07)	0.142
Did not go up stairs with support^c^ (*n* = 1,365)	15 (4.40)	11 (3.19)	0.71 (0.31–1.65)	6 (1.78)	0.39 (0.14–1.08)	14 (4.09)	0.93 (0.40–2.15)	0.634
Fine motor^c^								
Did not take off socks and shoes when asked to do so (*n* = 1,381)	58 (16.71)	60 (17.65)	1.00 (0.66–1.52)	61 (17.48)	0.95 (0.62–1.45)	54 (15.61)	0.82 (0.53–1.27)	0.356
Did not drink from an ordinary cup without help^d^ (*n* = 1,399)	6 (1.72)	5 (1.43)	0.83 (0.25–2.75)	4 (1.15)	0.66 (0.18–2.37)	4 (1.14)	0.66 (0.18–2.35)	0.464
Attention^c^								
Was not occupied alone with the same thing for at least 15 min (*n* = 1,381)	55 (15.99)	66 (19.08)	1.13 (0.75–1.69)	66 (19.13)	1.05 (0.69–1.59)	63 (18.16)	0.94 (0.61–1.44)	0.664
Cognition^c^								
Did not bring things when told to^d^ (*n* = 1,380)	4 (1.17)	3 (0.87)	0.74 (0.16–3.34)	3 (0.87)	0.74 (0.16–3.35)	6 (1.73)	1.49 (0.42–5.33)	0.274
Did not make marks on table or paper (*n* = 1,375)	12 (3.46)	14 (4.01)	1.00 (0.44–2.28)	22 (6.32)	1.51 (0.70–3.25)	23 (6.57)	1.21 (0.55–2.67)	0.464
Did not turn the picture right when looking in a book (*n* = 1,329)	174 (52.89)	175 (53.19)	1.00 (0.73–1.38)	151 (45.21)	0.71 (0.51–0.98)	177 (52.37)	0.94 (0.68–1.31)	0.345
Language^c^								
Did not use word-like sounds to tell what he/she wants (*n* = 1,380)	6 (1.72)	8 (2.29)	1.39 (0.46–4.25)	9 (2.58)	1.58 (0.51–4.91)	14 (3.98)	2.93 (1.00–8.56)	0.039
No. of things he/she can mention by name^e^ (*n* = 1,397)	Reference		0.78 (0.57–1.06)		0.98 (0.72–1.35)		0.88 (0.64–1.22)	0.797
Did not use sentences of two words (*n* = 1,357)	109 (71.71)	90 (59.21)	0.72 (0.52–1.00)	93 (59.24)	0.78 (0.56–1.08)	99 (60.74)	0.68 (0.49–0.95)	0.050

a HRs: time to event: the time from birth to the age when child (in months) can start sitting or walking alone.

b Adjusted for maternal age, parity, smoking and alcohol drinking during pregnancy, maternal education, maternal occupation, prepregnancy BMI, breast-feeding at child age 6 months, gestational weeks at blood drawing, out-of-home child care, how many hours together with mother per day, home density (rooms/person), sex.

c All the same covariates adjusted for the outcome of “sit” and “walk” plus child’s age at interview.

d Crude ORs because of sparse data.

e Ordinal logistic regression.
